# The Case for “Environment in All Policies”: Lessons from the “Health in All Policies” Approach in Public Health

**DOI:** 10.1289/EHP294

**Published:** 2016-06-28

**Authors:** Geoffrey R. Browne, Ian D. Rutherfurd

**Affiliations:** 1 McCaughey VicHealth Community Wellbeing Unit, Centre for Health Equity, School of Population and Global Health, and; 2 School of Geography, University of Melbourne, Parkville, Victoria, Australia

## Abstract

**Background:**

Both public health, and the health of the natural environment, are affected by policy decisions made across portfolios as diverse as finance, planning, transport, housing, education, and agriculture. A response to the interdependent character of public health has been the “health in all policies” (HiAP) approach.

**Objectives:**

With reference to parallels between health and environment, this paper argues that lessons from HiAP are useful for creating a new integrated environmental management approach termed “environment in all polices” (EiAP).

**Discussion:**

This paper covers the theoretical foundations of HiAP, which is based on an understanding that health is strongly socially determined. The paper then highlights how lessons learned from HiAP’s implementation in Finland, California, and South Australia might be applied to EiAP. It is too early to learn from evaluations of HiAP, but it is apparent that there is no single tool kit for its application. The properties that are likely to be necessary for an effective EiAP approach include a jurisdiction-specific approach, ongoing and strong leadership from a central agency, independent analysis, and a champion. We then apply these properties to Victoria (Australia) to demonstrate how EiAP might work.

**Conclusions:**

We encourage further exploration of the feasibility of EiAP as an approach that could make explicit the sometimes surprising environmental implications of a whole range of strategic policies.

**Citation:**

Browne GR, Rutherfurd ID. 2017. The case for “environment in all policies”: lessons from the “health in all policies” approach in public health. Environ Health Perspect 125:149–154; http://dx.doi.org/10.1289/EHP294

## Introduction

Some of the most important ‘environmental’ legislation does not lie within the administration of the Minister for Environment and Climate Change. It is in the hands of the central agencies such as Premier & Cabinet, Treasury, Planning, Public Transport and Roads & Ports, as well as others. Due to the interdependence of their portfolios with the natural environment, each of these ministers responsible should consider him or herself an ‘environment minister’ and their decisions should be made with due regard for natural systems (Commissioner for Environmental Sustainability 2008).

## Background

Since the 1970s, the most common way to ascertain the impacts of policy decisions on the environment has been through formal environmental impact assessments coordinated through the environment department of governments (Jay et al. 2007). However, the scale of environmental challenges and their impact on human wellbeing means that environmental impacts can no longer be viewed as only the domain of environment departments. Even policy proposals that do not have an immediate or obvious environmental element will often have long-term, unknown, or unintended environmental consequences (Grossman and Krueger 1991; Johnson 2001; Steinfeld et al. 2006). There is growing recognition that an approach is required that *a*) considers the environmental consequences of higher-level strategic policy (not just projects), and *b*) integrates consideration of environmental issues into the agendas of policy makers who do not typically consider the environment as their responsibility (Head et al. 2014).

Two approaches have been proposed in response: “integrated environmental management” (IEM) and “environmental policy integration” (EPI). IEM describes a holistic, intersectoral, and strategic approach to environmental management (Margerum 1997, 1999), whereas EPI is an approach intended to incorporate environmental objectives into each stage of policy development in nonenvironmental sectors such that the long-term environmental consequences of decisions are predicted and minimized (Eckerberg and Nilsson 2013; Lafferty and Hovden 2003; Nilsson and Persson 2003). Both IEM and EPI aim to reconcile the aims of development with the protection of ecosystem services by ensuring that all policy sectors are involved and accountable (Margerum 1999; Nilsson and Persson 2003). These approaches demonstrate recognition of the need, and some appetite, for comprehensive integration of environmental criteria into decision making at the highest levels. However, for IEM, there appears to be no definitive guidance on how integration should occur (Margerum 1999). Similarly, EPI is coherent as a concept but can be impractical to apply owing to political difficulties and the complexity of situations, and it has experienced challenges in effectively changing the way that policy decisions are made (Lafferty and Hovden 2003; Nilsson and Persson 2003). As a result, both approaches have had limited success in institutionalizing integrated environmental management such that essential ecosystem services are maintained (Rockström et al. 2009).

## Objectives

With reference to the parallels between health and environment, this paper argues that lessons from the current public health approach, “health in all policies” (HiAP), could be useful for creating a new integrated environmental management approach, “environment in all polices” (EiAP). HiAP explicitly asks policy makers in all areas to consider the health impacts of decisions. The approach is based on strong evidence that health is socially determined and that decision making in diverse policy areas, apparently unrelated to health, nevertheless affects health [Commission on Social Determinants of Health (CSDH) 2008; Marmot 2005; Rose 1992; Wilkinson and Marmot 2003]. The idea that social structures determine outcomes is mirrored in the understanding of environmental sustainability. Applied to health, social determinism stands in contrast to the individualistic approach of patient-centered medicine and the focus on health education and behavior change as a means of preventing illness (Bacigalupe et al. 2010). Applied to environmental sustainability, social determinism suggests that social infrastructure and policies from diverse sectors determine behavior (Grossman and Krueger 1991; Johnson 2001; Steinfeld et al. 2006), which creates impacts on the environment (Shove 2010). Advocates who hold this view acknowledge the limits of behavior change programs and state that both environment and public health practitioners should be policy—and indeed politically—active to improve respective determinants (Birn 2009; Nelson and Vucetich 2009).

There are several areas where environmental management has benefited from advances in public health [c.f. methods of systematic review (Roberts et al. 2006), the use of etiological approaches to describe environmental issues (Browne and McPhail 2011; Niemeijer and de Groot 2008), and advocates’ responses to the influential role of multinationals (Chan 2013; Gleeson and Friel 2013; Meckling 2011; Moodie et al. 2013)]. With reference to the socially determined nature of health and the environment, we explore whether lessons from the implementation of HiAP can be used to develop an EiAP approach. Recently, Varis et al. (2014) recognized the value that the HiAP approach can lend to natural resource management to suggest improvements to integrated water resources management. Here, we suggest that an EiAP approach would fulfill the ambitions of EPI and IEM to effectively place a “lens” over decision making at the policy development level to ask, “What will the environmental impacts of this policy be? Will there be unintended consequences? How can these be avoided, minimized, or at least made explicit?”

## Discussion

### The Foundations of HiAP: The Social Determinants of Health

**Figure 1:**
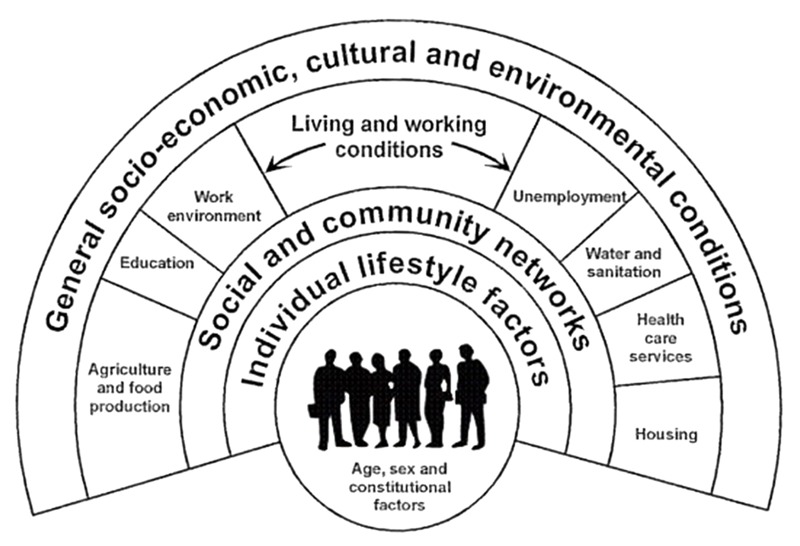
The determinants of health and well-being (Dahlgren and Whitehead 1991) (used with permission).

HiAP is founded in current models of population health that in turn borrow from ecology to suggest that health is the result of the way the structures of society interact with individuals (Lindström and Eriksson 2005). To develop effective interventions, ecological models of health explicitly consider how the multiple levels of society, the “causes of the causes” that lead to health, can be addressed (Rose 1992). Extensive research supports this ecological model and the proposition that the conditions under which we live, formed by policy (and politics), affect how healthy we are (Khaw and Marmot 2008; Marmot 2005; Sallis et al. 2008). The ecological model of health is encapsulated by the “social determinants of health” (SDH) framework (CSDH 2008; Wilkinson and Marmot 2003) and is illustrated in a well-known figure by Dahlgren and Whitehead (1991) (Figure 1).

If the natural, built, and social environments play a role in disease, then policy, and therefore politics, has a role to play in improving health and wellbeing (Birn 2009; Chan 2008; Friel and Denniss 2013; Marmot 2005; Pickett and Wilkinson 2010). Indeed, as Marmot and Bell (2012) assert, because “the major determinants of health are social, so must be the remedies,” much poor health is preventable, and all public policy sectors have a role to play in that prevention, not only the health sector.

### From SDH to Health in All Policies

The need for public policy that benefits health was first recognized in the 1986 Ottawa Charter for Health Promotion in the phrase “healthy public policy” [World Health Organization (WHO) 1986]. It was born out of an understanding of the SDH, recognition of the necessity of intersectoral action on health, plus approaches to assessment of the impact of major projects (i.e., Health Impact Assessment; HIA) (Collins and Koplan 2009; Ståhl et al. 2006). However, it is likely that the catchphrases in use at the time (c.f. “healthy public policy”) did not “speak” to policy makers in the intended manner. It was during the second Finnish presidency of the European Union 20 years later, that the hortatory phrase, “terveys kaikissa politiikoissa” (literally “health in all policies”) arose. It had linguistic strength compared with previous phrases and encapsulated the Finnish contribution to the advancement of intersectoral action for health. In line with attempts to rebuild confidence in the ability of governments to improve health in the EU, the HiAP approach was intended to address social determinants and to “move health higher up the European agenda” (Ståhl et al. 2006).

The concept was further endorsed in 2007, in Article 152 of the European Union Treaty, which stated that a “…high level of human health protection shall be ensured in the definition and implementation of all community policies and activities...” (European Community 2007). Following the Rome Declaration on HiAP in 2007 (Health Ministerial Delegations of EU Member States 2007) and the Adelaide Statement on HiAP in 2010 (McQueen et al. 2012), a consensus definition of HiAP was adopted in 2013 at the conclusion of the 8th Global Conference on Health Promotion in Helsinki:

*Health in All Policies* is an approach to public policies across sectors that systematically takes into account the health implications of decisions, seeks synergies, and avoids harmful health impacts in order to improve population health and health equity. It improves accountability of policymakers for health impacts at all levels of policy-making. It includes an emphasis on the consequences of public policies on health systems, determinants of health and well-being (WHO 2013).

There has been considerable international activity under the catchphrase of HiAP, with adoption of a version of the approach in ≥ 16 countries at the national or state-equivalent level (Baum et al. 2014; Greaves and Bialystok 2011; Health in All Policies Task Force 2010; St-Pierre 2008), and it has gained traction in strategic health planning, even at the local government level (Department of Human Services, State Government of Victoria 2001; Rudolph et al. 2013a; Public Health and Wellbeing Act 2008). Finland, (Kickbusch 2010; Melkas 2013; Puska and Ståhl 2010; St-Pierre 2008), California [Health in All Policies Task Force 2010; Rudolph et al. 2013b; California Strategic Growth Council (SGC) 2015], and British Columbia (Greaves and Bialystok 2011; Geneau et al. 2009) are notable for their development, application, and documentation of the approach. Similarly, its application in South Australia (SA) is particularly instructive for a proposed EiAP (Flinders University 2013; Kickbusch et al. 2014; Lawless et al. 2012; SA Health 2011, 2012b).

### Applying Lessons from HiAP to EiAP

In the context of much professional enthusiasm for HiAP, there has been relatively little evaluation, partly because HiAP is quite new and evaluation methodologies are not yet well formed (Greaves and Bialystok 2011). Further, HiAP’s ambition to address health via social determinants is a necessarily complex task (Baum et al. 2014; Butland et al. 2007), which is likely to make attribution of any improvement in population health to HiAP difficult. In response to this challenge, Bauman et al. (2014) proposed a form of “complex contribution analysis” to estimate and model the intended impacts of HiAP and to compare the findings with the results of empirical evaluations when they become available. In contrast, Baum et al. (2014) proposed a more sociological approach to evaluation, recommending that a “burden of evidence” is sufficient to support logically coherent chains of effectiveness. At the time of writing, these approaches have not been trialed, but results from such evaluations would be valuable information from which to implement EiAP.

Nevertheless, a synthesis of the literature about implementation of HiAP in the regions where it has been implemented provides useful lessons. These are summarized and then applied to the way in which EiAP might be implemented in Victoria, Australia in the next section.

A principal lesson for EiAP is that although HiAP is coherent in concept, there is no single tool kit for its implementation (Greaves and Bialystok 2011; Rudolph et al. 2013a). Rather, the take-up of HiAP ranges from the adoption of general policy positions to specific decision-making procedures and mechanisms that model the health consequences of policy and then respond to them (Puska and Ståhl 2010; SGC 2015), such as the health lens analysis in SA (Delaney et al. 2015; SA Health 2011, 2012a). This suggests that an effective EiAP approach will require new jurisdiction-specific structures and processes to ensure that environmental criteria permeate meaningfully into decision making (Eckerberg and Nilsson 2013; Lane and Robinson 2009; McQueen et al. 2012).

The implementation of HiAP also indicates that for EiAP, the challenges of incorporating environmental criteria into areas not traditionally accustomed to their consideration should not be underestimated (Nilsson and Persson 2003), particularly in the current political climate (Bacigalupe et al. 2010; Konisky et al. 2008). As Greaves and Bialystok (2011) found of HiAP, implementation of EiAP will require public service leaders across multiple, diverse portfolios to “rise above their own interests, consider shared goals and commit to steps for reaching them.” These authors state that the short election cycle, the compartmentalized character of bureaucracy, and the lack of effective tools for identifying the health impact of nonhealth policies are also challenges. In SA, these challenges are dealt with via bipartisan mandate from State government and a dedicated centrally governed HiAP unit that is tasked with supporting independent analysis of policies’ effects on health (Delaney et al. 2015; SA Health 2012a). Another challenge of HiAP is that health is not unique in its need for a mechanism that cuts across government silos (Flinders University 2013). Many sectors believe their own policy area to be unique and would benefit from integration, and the use of HiAP has been criticized for attempting to legitimize the securing of scarce resources (Pinto et al. 2015). Although any attempt at EiAP must avoid accusations of “environmental imperialism” (Kemm 2001), the natural environment is the ultimate provider of services essential to life (Costanza et al. 1997; Watts et al. 2015; Millennium Ecosystem Assessment 2005), and arguably warrants special attention. Further, placing EiAP processes with central agencies with authority (e.g., the Department of Premier and Cabinet—see example below), as has been done elsewhere, should avoid such accusations. Nevertheless, any attempt at EiAP should be approached sensitively lest it alienate colleagues from other policy areas.

To address the abovementioned challenges, successful implementation of EiAP is likely to require the alignment of a number of conditions, actions, and structures (McQueen et al. 2012). Kickbusch et al. (2014) argue that HiAP gained traction in SA because of a serendipitous alignment of conducive governance structure, leadership from a central agency, policy heritage, and the timing of the State’s Strategic Plan. Similarly, Greaves and Bialystok (2011) claim that a major crisis or initiative is required to trigger a move to HiAP, citing the example of British Columbia and the 2010 Olympic Games. In this case, the aim of “making British Columbia the healthiest jurisdiction ever to host,” granted the government enough support to launch the HiAP approach “ActNow,” which had steady and high-level leadership and momentum, even when all the elements or the ideal conditions were not in place (Geneau et al. 2009).

Positioning EiAP

**Figure 2:**
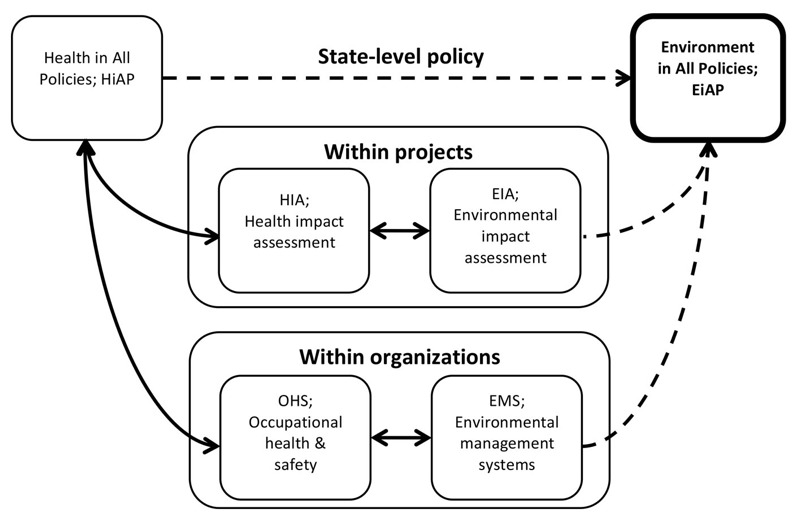
An “environment in all policies” (EiAP) approach complements existing environmental management tools at other levels, as “health in all policies” (HiAP) does for health. Solid lines show how existing approaches are informed by each other, and dashed lines show how EiAP would be informed by existing approaches.

The HiAP rhetoric has arguably enhanced the understanding that health is socially determined. It has created a discourse that has sensitized decision makers in diverse policy areas to the need to account for, or at least make explicit, the impacts on health of their policy decisions. The practice of HiAP therefore provides support for the idea of EiAP that would fulfill the ambitions of EPI and IEM. It would also complement existing environmental management tools at other levels, as HiAP does for health [c.f. HiAP, HIA, EIA, occupational health and safety (OHS), and environmental management systems (EMS) (Beckmerhagen et al. 2003; International Association for Impact Assessment 1999; WHO 2014)](Figure 2). An effective EiAP approach would not only encourage governments and bureaucracies to consider the environment at all stages of decision making but also force them to *a*) make explicit the magnitude of known consequences of strategic-level policy options and *b*) identify unintended environmental consequences of those options. As shown for HiAP, with the aid of a “champion” (Rudolph et al. 2013a), as well as a defined, jurisdictionally appropriate process, EiAP will enhance the way that policy development considers and minimizes environmental impacts. Exactly how EiAP would operate would vary across jurisdictions, but we propose the following principles:

EiAP should sit at a higher level than environmental impact assessments, that is to say, at the level of major policy.
EiAP is most critically applied at the scale of provincial or state governments rather than local or national levels.
EiAP should operate at the level of cabinet decisions, providing reviews of the environmental consequences of policy options being considered.
EiAP reviews must be subject to independent analysis and, if possible, be made public (although this can be difficult at the level of confidential cabinet discussions).
An EiAP champion with significant existing influence should be appointed and tasked with “socializing” the approach across government and facilitating the process at the operational level.

### Possible Model of EiAP: An Environmental Bill of Rights

Currently, no examples of such an EiAP approach exist. A close example is the Ontario (Canada) Environmental Bill of Rights (EBR) (1993). Under the EBR legislation, 15 government ministries have to produce a Statement of Environmental Values (SEV) document. Each Minister must ensure that the SEV is considered whenever decisions that might significantly affect the environment are made. The EBR is administered by an independent Environment Commissioner. Environmentally significant acts, regulations and policies must be posted to an environmental registry. The public is also empowered by the EBR to review and challenge the posted proposals.

This legislation is now > 20 years old, and components of this legislation support an EiAP approach. Importantly, the system is founded on *a*) an articulation, across all parts of government, of environmental values and how decisions likely to affect the environment will be made; *b*) communication of major pending decisions via a registry; *c*) clear powers and ways for the community to challenge decisions; and *d*) an independent entity to regulate the process (Environmental Bill of Rights 1993). In contrast to the principles we described above, the EBR relates to departmental actions rather than to higher-level cabinet decisions: Most of the examples in the environmental registry are specific projects or planning proposals that then attract comment from the public.

### Example Model of EiAP: A Cabinet Approach

Building on this example, we propose a two-stage process to lead to EiAP, using the government of the state of Victoria (Australia) as an example:

Stage 1
Review of policy should take place at the genesis of major reforms, that is, at the level of cabinet proposals. Because major departmental initiatives (such as legislative reviews or major policy shifts) always go through the cabinet, this is the appropriate point of review.
The proposal would be scrutinized for environmental consequences before it is considered by the cabinet (i.e., all major policy would have an “environmental consequence” addendum). This process would be called a “preliminary review.” The purpose of the preliminary review is to explicitly identify obvious environmental issues early, before commitments are made to proceed.
Stage 2
Next, a more comprehensive environmental assessment, such as an environmental lens analysis (ELA; analogous to the health lens analysis in SA), should be coordinated through the government leader’s office rather than through any particular portfolio. In Victoria, the appropriate organization in state government would be the Office of Premier and Cabinet. Ideally, the review would be made public to build confidence in the process and its recommendations, but this would depend on the cabinet process.
A review of the lens analysis for its findings, as well as for its adequacy, should be performed by an independent entity, such as an Environment Commissioner. There is a Commissioner for Environmental Sustainability in Victoria, but the role of this commissioner is to review the state of the environment, rather than to review programs, so this role would need to be extended.
As proposals progress through the cabinet, the results of the lens analysis would be used to make explicit and to minimize the environmental impacts of the process (whether intended or unintended). As a result, legislation could be developed or altered, or a “White Paper” leading to new policy could be prepared.

An analogue to this process is the Regulatory Impact Statement (RIS), which is required to accompany any new (or sunsetting) policy or legislation in Victoria under the Subordinate Legislation Act 1994 (1994) (s.7). An RIS, prepared by the relevant Minister, must assess the impacts of the policy change in terms of the “triple bottom line.” The quality of the RIS is then reviewed by the independent Victorian Competition and Efficiency Commission with the intention that outcomes of the RIS improve the policy or legislation.

## Conclusion

Discourse on the integration of environmental policy has recognized that there is a need to elevate consideration of the environmental effects of decision making if essential ecosystem services are to be sustained, but to date, there has been limited success when applying these approaches. There are many similarities between the environment and public health, where a major initiative has been HiAP. We believe that, informed by lessons learned from the implementation of HiAP, there is an opportunity to develop an EiAP approach in government to meet this need. The benefit of integrated policy making as exemplified by HiAP and the proposed EiAP is that it has the potential to act upon the social determinants of population health and environmental health, respectively, to make critical (and potentially unavoidable) trade-offs between environment, public health, and economic priorities transparent; to improve decision making; and can help to create a more sustainable society. Reviews of the implementation of HiAP show that although there have been challenges and no single method of implementation, it has proved promising in its aim of integration across portfolios for the benefit of public health. We propose that there is scope for an EiAP approach to operate at a similar level to that of HiAP, such as that of the cabinet, at the scale of state- or provincial-level decisions, and we welcome further discussion and refinement of the proposal.
